# A short-term, randomized, controlled, feasibility study of the effects of different vegetables on the gut microbiota and microRNA expression in infants

**DOI:** 10.3389/frmbi.2024.1342464

**Published:** 2024-03-01

**Authors:** Lynn E. Ferro, Kyle Bittinger, Sabrina P. Trudo, Kaleigh E. Beane, Shawn W. Polson, Jae Kyeom Kim, Jillian C. Trabulsi

**Affiliations:** ^1^ Department of Health Behavior and Nutrition Sciences, University of Delaware, Newark, DE, United States; ^2^ Division of Gastroenterology, Hepatology, and Nutrition, Children’s Hospital of Philadelphia, Philadelphia, PA, United States; ^3^ School of Human Environmental Sciences, University of Arkansas, Fayetteville, AR, United States; ^4^ Center for Bioinformatics and Computational Biology, University of Delaware, Newark, DE, United States; ^5^ Department of Food and Biotechnology, Korea University, Sejong, Republic of Korea

**Keywords:** Apiaceae, broccoli, carrot, Cruciferae, infant feeding, microbiome

## Abstract

The complementary diet influences the gastrointestinal (gut) microbiota composition and, in turn, host health and, potentially, microRNA (miRNA) expression. This study aimed to assess the feasibility of altering the gut microbial communities with short-term food introduction and to determine the effects of different vegetables on the gut microbiota and miRNA expression in infants. A total of 11 infants were randomized to one of the following intervention arms: control, broccoli, or carrot. The control group maintained the milk diet only, while the other groups consumed either a broccoli puree or a carrot puree on days 1–3 along with their milk diet (human milk or infant formula). Genomic DNA and total RNA were extracted from fecal samples to determine the microbiota composition and miRNA expression. Short-term feeding of both broccoli and carrots resulted in changes in the microbiota and miRNA expression. Compared to the control, a trend toward a decrease in Shannon index was observed in the carrot group on days 2 and 4. The carrot and broccoli groups differed by weighted UniFrac. *Streptococcus* was increased on day 4 in the carrot group compared to the control. The expression of two miRNAs (i.e., miR-217 and miR-590-5p) trended towards decrease in both the broccoli and carrot groups compared to the control, whereas increases in eight and two different miRNAs were observed in the carrot and broccoli groups, respectively. Vegetable interventions differentially impacted the gut microbiota and miRNA expression, which may be a mechanism by which total vegetable intake and variety are associated with reduced disease risk.

## 1 Introduction

During infancy, the gut microbiome is in its development stage. Early factors such as the mode of delivery, use of antibiotics, the liquid diet in infancy (i.e., human milk and/or infant formula), and the introduction of complementary foods, both the timing and the composition, result in differentiated gut microbial profiles (Robertson et al., 2019). These factors affect the colonization of intestinal bacteria, which in turn affects the development and physiology of the early-life host immune system, with implications for later-life health and disease (Tanaka and Nakayama, 2017). The gut microbiome becomes more stable with age, reaching an adult-like state around 2–3 years of age, rendering infancy a sensitive period for the establishment of a diverse collection of microbes (Deering et al., 2019). At the time of weaning and subsequent introduction to solid foods, the infant gut microbiota transitions toward one dominant in the phyla Bacteroidetes and Firmicutes (Tanaka and Nakayama, 2017). While the relationship between diet and the microbiota has been studied in adult populations, particularly diet patterns and disease risk (Chen et al., 2014; Tindall et al., 2018), and there is a general understanding of changes in the microbiota composition with solid food introduction (Laursen et al., 2017), there is a paucity of data, particularly randomized controlled trials (RCTs), evaluating the impact of individual solid foods on the developing infant gut microbiota. Of the RCTs that have been conducted in infancy, only a few have determined the impact of singular solid foods (infant cereal, legumes, or meat) on gut microbial communities (Krebs et al., 2013; Ordiz et al., 2020; Plaza-Diaz et al., 2021; Qasem et al., 2017).

MicroRNAs (miRNAs) are non-coding single-stranded RNA, approximately 22 nucleotides in length, that function as regulators in host gene expression processes (Ionescu et al., 2022). Fecal miRNAs are considered biomarkers of intestinal diseases, and recent studies have suggested that miRNAs produced by intestinal epithelial cells of the host play a role in shaping the gut microbiota by stimulating or inhibiting the growth of certain bacterial species (Ionescu et al., 2022; Dalmasso et al., 2010). The specific molecular mechanisms remain unknown; however, miRNAs affect the barrier function of intestinal cells. Some studies have shown that the microbiota can in turn affect miRNAs, modulating the expression of host genes (Ionescu et al., 2022). Diet also influences miRNA expression, which has primarily been studied in the context of disease, particularly cardiovascular disease and cancers (Palmer et al., 2014; Otsuka and Ochiya, 2021; Kura et al., 2019). Dietary constituents can modulate miRNA expression levels, which in turn regulate mRNAs involved in lipid metabolism, inflammation, apoptosis, and cell migration (Cui et al., 2017). More specifically, polyunsaturated fatty acids (PUFAs) have been shown to downregulate miR-146a and miR-21, which play roles in the induction of vascular inflammation (Roessler et al., 2017; Hulsmans et al., 2011) and the reduction of pro-inflammatory signaling (Palmer et al., 2014), respectively. Another example is the downregulation of miR-146a and miR-155 by vitamin D in murine models, which in turn inhibits NF-kB (nuclear factor kappa light-chain enhancer of activated B cells) and can lead to the suppression of inflammation (Karkeni et al., 2018).

More recently, apiaceous and cruciferous vegetables, more commonly known as carrot and broccoli family vegetables, respectively, have been studied for their impact on miRNA expression in animal models (Kim et al., 2019; Kim et al., 2018). Both apiaceous and cruciferous vegetables have anti-inflammatory and anti-cytotoxic properties (Kobaek-Larsen et al., 2019; Tilg, 2015; Costa-Pérez et al., 2023). Apiaceous vegetables contain the bioactive polyacetylenic oxylipins falcarinol and falcarindiol (Kobaek-Larsen et al., 2019), while cruciferous vegetables contain glucosinolates and form biologically active compounds such as indoles and isothiocyanates (Tilg, 2015; Costa-Pérez et al., 2023). With regard to the microbiota, bacteria are able to digest both polyacetylenes and glucosinolates and in turn alter the composition of the gut microbiota (Wu et al., 2019; Li et al., 2009; Trudo et al., 2020); however, little research has examined the effect of these vegetable families, which are commonly consumed, on the gut microbiota and miRNA expression in humans. More specifically, infants commonly consume vegetable purees as one of the first solid foods, but the literature on the effect of singular foods and their impact on the microbiota and miRNA expression is limited. Moreover, the interrelationships among diet, microbiome, and miRNA have seldom been studied. One large-scale study in adults examined the association of diet patterns (i.e., vegan, vegetarian, and omnivore) with fecal miRNA expression and epigenetic state and the gut microbial community composition and was found that stool miRNA profiles were associated with the diet patterns, particularly altering the levels of miRNAs related to lipid metabolism, which correlated with the relative abundance of *Akkermansia muciniphila* (Tarallo et al., 2022).

With limited research examining diet as a modulator of both miRNA and the microbiome, coupled with the knowledge that infancy is a sensitive period for growth and development, the present study aimed to determine the feasibility of the short-term feeding of an apiaceous and a cruciferous vegetable on the gut microbiota and miRNA expression and to evaluate preliminary relationships between the microbiota and miRNA expression of 6-month-old infants.

## 2 Materials and methods

### 2.1 Study design

In this randomized, controlled feasibility study, participants were recruited from the Northwest Arkansas region from August 2018 through August 2019. The inclusion criteria were: infants who were 6 months old at the time of enrollment; parents who were willing to avoid over-the-counter and/or prescription medications for their infant and exclude the feeding of all fruits, vegetables, and herbs to their infant from 10 days prior to the start of the study until the conclusion of the study; compliance with the study protocol; and having no plans to travel during the study duration. The exclusion criteria were as follows: infants with liver, kidney, or intestinal disorder history; use of prescription or over-the-counter medications; and known allergies or intolerances to the intervention foods, namely, broccoli or carrot. This study was reviewed and approved by the University of Arkansas Institutional Review Board. Parental consent was obtained for all participants.

A total of 14 infants were enrolled. Two participants dropped out prior to the start of the study and one participant did not provide fecal samples, resulting in a total sample size of 11. Infants were randomized to one of three groups: control (*n* = 3), carrot (*n* = 4), or broccoli (*n* = 4). The carrot puree consisted of carrots, purified water, and olive oil, while the broccoli puree consisted of broccoli and purified water (Harvest to Highchair, Mt. Pleasant, SC, USA). On study days 1, 2, and 3, infants consumed 56.7 g (2 oz.) per day of their randomization-assigned study food while continuing their liquid milk diet (infant formula and/or human milk). Infants in the control group continued their liquid milk diet only. In addition to recording the intake of the assigned study food each day of the intervention period, parents/legal guardians collected fecal samples throughout the study.

### 2.2 Fecal sample collection

Fecal samples were collected on days 0 (baseline), 2, 4, and 6. Samples were collected in the diaper, placed in a sealed bag, stored in the participant’s home freezer, and collected by research personnel within 24 h. The samples were transported frozen to the laboratory and stored at −80°C until preparation for analysis.

### 2.3 Microbiota assessment

Fecal genomic DNA (gDNA) from days 0, 2, 4, and 6 was extracted from fecal samples using the DNeasy PowerSoil Pro Kit (Qiagen, Hilden, Germany) and following the manufacturer’s instructions. The gDNA concentrations were quantified using the Qubit 3.0 Fluorometer (Thermo Fisher Scientific Inc., Waltham, MA, USA). Of the resulting gDNA, 5–10 ng was transferred to corresponding wells in a PCR plate. A 2× PCR mix was added, and PCR was performed as per the manufacturer’s instructions to amplify and barcode each sample. The samples were purified via spin column using the MinElute PCR Purification Kit (Qiagen, Hilden, Germany). The resultant 16S/ITS/23S amplicon pool (~2,500 bp) was used as a template to construct an SMRTbell library using Express Template Prep 2.0 (Pacific Biosciences, Menlo Park, CA, USA) as per the manufacturer’s Template Preparation protocol. The quality of the amplicon library was assessed using the Qubit 3.0 Fluorometer (Thermo Fisher Scientific Inc., Waltham, MA, USA) and the Agilent Femto Pulse System (Agilent Technologies, Santa Clara, CA, USA). The amplicon library was run on a single Sequel IIe system 8M SMRT cell using sequencing chemistry 3.0 with 4 h pre-extension and 30 h movie time.

### 2.4 miRNA expression assessment

Fecal RNA samples from days 0 and 4 were isolated using the RNeasy PowerMicrobiome kit (Qiagen, Hilden, Germany) following the manufacturer’s instructions. The quality of isolated RNA was assessed using the A260/280 and A260/230 ratio measurements (SpectraMax i3x; Molecular Devices, Sunnyvale, CA, USA). RNA samples were stored at −80°C until profiling.

The extracted fecal RNA was normalized to a single concentration (33 ng/µl) and loaded into 96-well plates. Fecal miRNA profiling using the NanoString nCounter platform (NanoString, Seattle, WA, USA) was performed at the University of Minnesota Genomic Center (Minneapolis, MN, USA). The pre-built panel for human miRNA (nCounter Human v3 miRNA panel) was used, in which a total of 827 different miRNAs were analyzed.

### 2.5 Bioinformatics and statistical analyses

Between-group differences in the characteristics of the participants were assessed using analysis of variance (ANOVA) for continuous variables and Fisher’s exact test for categorical variables.

Sequence data were demultiplexed and filtered using SBPipe (ver. 3.1.R38b5a8f; Shoreline Biome, Farmington, CT, USA). The denoising and clustering of data into amplicon sequence variants (ASVs) was performed in DADA2 (ver. 1.18.0) (Callahan et al., 2016) via the Shoreline Biome DADA2_R_pipeline.R with OMEGAA 1e-10 and OMEGAC 1e-80. Taxonomy was assigned to ASVs using SBPipe against a merged Athena (ver. 2.2) and SILVA (ver. 138) (Yilmaz et al., 2014) database. The taxonomy and ASV tables were imported into QIIME2 (ver. 2021.4) (Bolyen et al., 2019) for data visualization and further processing. The ASV table was filtered to remove ASVs that had a combined count <3 or were represented in <2 samples. Analysis of the taxonomic composition was performed using ANCOM (Mandal et al., 2015). Sequences were aligned using MAFFT (Katoh et al., 2002), and an approximate maximum likelihood tree was constructed using FastTree with posterior midpoint rooting (Price et al., 2009). Alpha and beta diversity analyses were rarefied by subsampling 1,759 sequences, except for compositional statistical tests that utilized complete data normalized using centered log ratio after zero replacement with a pseudocount of 1 (Aitchison et al., 2000). Alpha diversity was assessed using the Shannon index and observed ASVs (Faith, 1992). Beta diversity analysis examined weighted (Lozupone et al., 2007) and unweighted (Lozupone and Knight, 2005) UniFrac distances and was visualized using principal coordinate analyses (PCoA).

Linear mixed-effects modeling was used to determine the effect of the treatment group (control, broccoli, or carrot), time (day 0, 2, 4, or 6), and group × time interaction on the a) relative abundance of bacteria at the genus and species levels (where the mean relative abundance was greater than 1% across groups) and the b) alpha diversity indices Shannon index and observed ASVs. The relative abundance of bacteria at each taxonomic level was log_10_ transformed in the linear mixed-effects model. Permutational multivariate analysis of variance (PERMANOVA) was used to assess for beta diversity indices weighted and unweighted UniFrac distances by group. *P*-values were adjusted due to multiplicity, where necessary, using the Benjamini–Hochberg false discovery rate.

The miRNA expression data were normalized to baseline (day 0). The effect of the feeding group (control, broccoli, or carrot) over time (day 0 or 4) on the miRNA expression level was assessed using a linear model. Only miRNAs with a count ≥7.5 and a fold change (intervention groups compared to the control) ±1.5 were included in the linear model. miRNAs that were differentially expressed by feeding group were evaluated with Ingenuity Pathway Analysis (IPA) software to predict potential mRNA targets, which were then subjected to Protein Analysis Through Evolutionary Relationships software (PANTHER; available at www.pantherdb.org) to assess predicted overrepresented pathways.

The miRNAs were tested for normality using the Shapiro–Wilk test. Exploratory correlation analyses of the miRNAs differentially expressed among feeding groups and the taxon relative abundance (at the genus and species levels) were conducted using Spearman’s rank correlation. Only taxa present in more than one infant on day 0 or 4 were included. Due to the exploratory nature of this analysis and the small study sample size, all participants, regardless of diet intervention, were combined and correlational *p*-values are presented without correction.

The significance level for all analyses was set at *α* = 0.05. All analyses and visualizations were conducted using R software 4.2.1 with the packages adonisplus, broom.mixed, haven, lmertest, and tidyverse.

## 3 Results

### 3.1 Baseline characteristics of the participants

The characteristics of the participants are presented in **Supplementary Table 1**, which did not differ between groups at baseline. Briefly, the mean age of participants was 6.14 months, with 45% of the sample being males and 73% of the sample consuming a liquid diet that contained human milk. Moreover, 45% of the infants also had exposure to cereals prior to the study.

### 3.2 Microbiota

**Figure 1:**
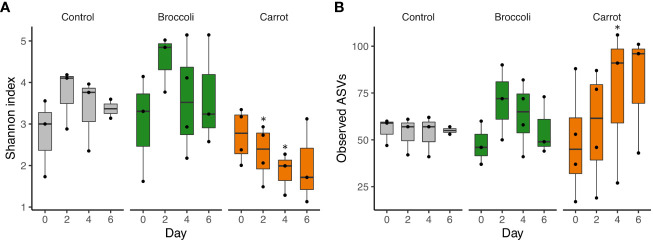
Alpha diversity. **(A)** Shannon index. The carrot group, compared to the control group, had a trend toward a decrease in Shannon index on days 2 and 4 (*p* = 0.06 and *p* = 0.09, respectively), while the carrot group, compared to the broccoli group, also had a decrease in Shannon index on day 2 (*p* = 0.02) and trended toward a decrease on day 4 (*p* = 0.08). **(B)** Observed amplicon sequence variants (ASVs). The carrot group, compared to the control group, trended toward an increase in the observed ASVs on day 4 (*p* = 0.11), but no differences were observed for the carrot group compared to the broccoli group (all *p* > 0.5). All time points, with the exception of day 6 in the control group, had at least three samples. ^∗^ *p* ≤ 0.1 (trend); ^∗∗^ *p* ≤ 0.05 (significance).

**Figure 2:**
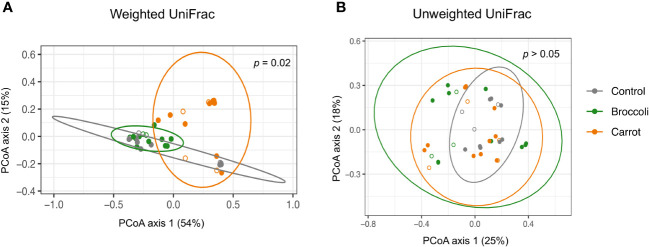
Beta diversity. **(A)** Carrot and broccoli samples differing by weighted UniFrac distance (*p* = 0.02). **(B)** Significant group effects were not observed for unweighted UniFrac distance (*p* > 0.05). *Open circles* denote day 0 (baseline); *filled circles* denote days 2, 4, and 6.

The short-term feeding of both broccoli and carrots resulted in changes in the alpha diversity, beta diversity, and relative abundance by group and time, which underscores the successful feasibility of this short-term feeding study. Firstly, alpha diversity was investigated to evaluate the within-sample diversity (evenness and number of bacteria) in the gastrointestinal community. A trend toward a decrease in the Shannon index was observed in the carrot group compared to the control group on days 2 and 4 (*p* = 0.06 and *p* = 0.09, respectively) and in the carrot group compared to the broccoli group on days 2 and 4 (*p* = 0.02 and *p* = 0.08, respectively) (**Figure 1**). No differences were found for the Shannon index in the broccoli group compared to the control group (*p* > 0.05). There was a trend for an increase in the observed ASVs in the carrot group compared to the control on day 4 only (*p* = 0.11). No differences were found for the observed ASVs between the broccoli and control groups or the carrot and broccoli groups. Subsequently, beta diversity, a measure of similarity or dissimilarity between samples, was evaluated. The weighted UniFrac distances differed between the carrot and broccoli groups (*p* = 0.02) (**Figure 2**). There were no significant group effects observed for the unweighted UniFrac distances (*p* > 0.05) and no significant time or group × time effects for both weighted and unweighted UniFrac distances (*p* > 0.05).

**Figure 3:**
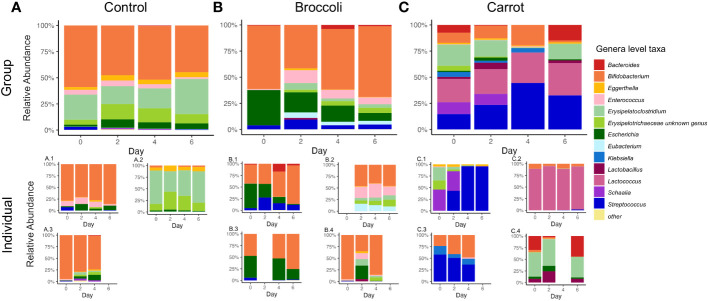
Relative abundance of the top microbial genera in the control **(A)**, broccoli **(B)**, and carrot **(C)** groups on days 0, 2, 4, and 6. In the carrot group compared to the control, *Streptococcus* was increased on day 4 (*p* = 0.03), while in the carrot group compared to broccoli, *Streptococcus* was increased on day 6 and trended toward an increase on day 4 (*p* = 0.02 and *p* = 0.06, respectively). In the carrot group compared to broccoli on day 6, *Erysipelatoclostridium* was increased (*p* = 0.04). Below each group stacked bar chart are stacked bar charts for each individual in the group.

Finally, the relative abundance of taxa was determined to quantify the gastrointestinal bacterial composition. The top 13 genera and 17 species had mean relative abundances greater than 1% across groups. The most abundant genera of the bacterial taxa found in the microbiota of the 11 six-month-old infants in this study were *Bifidobacterium*, *Streptococcus*, *Erysipelatoclostridium*, *Lactococcus*, and *Escherichia.* Significant group × time interactions were observed for *Streptococcus*, *Erysipelatoclostridium*, and *Enterococcus gallinarum.* In the carrot group, *Streptococcus* was increased on day 4 compared to the control (*p* = 0.03) and on day 6 compared to broccoli (*p* = 0.02). There was also a trend toward increased *Streptococcus* on day 4 compared to broccoli (*p* = 0.06) (**Figure 3**; also see **Supplementary Table 2**). In the carrot group compared to broccoli, *Erysipelatoclostridium* was increased on day 6 (*p* = 0.04). In the carrot group, *E. gallinarum* was decreased on day 2 compared to broccoli (*p* = 0.02) (**Supplementary Figure 1**; also see **Supplementary Table 2**). Group effects between the carrot and broccoli groups, but not a group × time interaction, were observed for *Bifidobacterium longum* and *Escherichia coli*. Both *B. longum* and *E. coli* trended towards a decrease in the carrot group compared to the control (both *p* = 0.10).

### 3.3 miRNA

The short-term feeding of both broccoli and carrot showed alterations compared to the control in miRNA expression over time (day 0 to day 4), thus providing encouraging evidence of feasibility. There were 10 miRNAs with a detection count ≥7.5 and a fold change ±1.5 that differed between the carrot and control groups. There were increases and trends toward increases in miR-143-3p, miR-186-5p, miR-345-3p, miR-448, miR-612, miR-769-5p, miR-1253, and miR-3144-3p (*p* = 0.10, 0.07, 0.08, 0.04, 0.03, 0.06, 0.08, 0.02, and respectively) and a decrease in miR-217 and miR-590-5p (*p* = 0.02 and *p* = 0.05, respectively) in the carrot group compared to the control. There were four miRNAs with a detection count ≥7.5 and a fold change ±1.5 that differed between the broccoli and control groups. There was a trend towards increase in miR-362-3p and miR-612 (*p*=0.06 and *p*=0.07, respectively) and a decrease in miR-590-5p and trend towards decrease in miR-217 (*p*=0.02 and *p*=0.06, respectively) in the broccoli group compared to the control. Of the 10 miRNAs that were differentially expressed in the carrot group compared to the control, miR-143-3p, miR-186-5p, miR-217, miR-590-5p, and miR-612 collectively had 60 predicted downstream target mRNAs (**Table 1**), with the expression of miR-590-5p and miR-143-3p contributing the majority of targets (70% and 11%, respectively). Of the four miRNAs that were differentially expressed in the broccoli group compared to the control, miR-217, miR-590-5p, and miR-612 collectively predicted 45 downstream target mRNAs (**Table 1**). The expression of miR-590-5p contributed the majority (93%) of the predicted targets.

**Table 1:** Effects of carrot and broccoli compared to control on the miRNA expression and target mRNA prediction.

	miRNA symbol	miRNA base accession	Fold change	*p*-value	Target mRNA^a^
Carrot	hsa-miR-143-3p	MIMAT0000435	1.7	0.1	*BCL2*, *DMNT3A*, *FNDC3B*, *IGFBP5*, *KRAS*, *MAPK12*, *MAPK7*, *MDM2*, *PLK1*, *PRC1*, *TOP2A*
	hsa-miR-186-5p	MIMAT0000456	2.15	0.07	*DDX43*, *FOXO1*, *HMGA2*, *TWIST1*
	hsa-miR-217	MIMAT0000274	−2.6	0.02	*TRPS1*
	hsa-miR-345-3p	MIMAT0022698	1.5	0.08	Not predicted
	hsa-miR-448	MIMAT0001532	4.86	0.04	Not predicted
	hsa-miR-590-5p	MIMAT0003258	−2.6	0.05	*ACTA2*, *APAF1*, *BMPR2*, *BTG2*, *C8orf44-SGK3/SGK3*, *CDC25A*, *CDK6*, *CFKN1A*, *CFL2*, *FAM3C*, *FAS*, *FASLG*, *GLCCI1*, *HIPK3*, *IL6R*, *JAG1*, *LRRFIP1*, *MARCKS*, *MTAP*, *NFIB*, *PDCD4*, *PELI1*, *PIK3R1*, *PRRG4*, *PTEN*, *RECK*, *RP2*, *SERPINB5*, *SESN1*, *SLC12A1*, *SLC16A10*, *SOCS5*, *SOD3*, *SOX5*, *SPRY1*, *SPRY2*, *TAGLN*, *TCF21*, *TGFBR2*, *TIMP3*, *TNF*, *TPM1*
	hsa-miR-612	MIMAT0003280	1.85	0.03	*AKT2*, *TP53*
	hsa-miR-769-5p	MIMAT0003886	2.65	0.06	Not predicted
	hsa-miR-1253	MIMAT0005904	1.65	0.08	Not predicted
	hsa-miR-3144-3p	MIMAT0015015	1.98	0.02	Not predicted
Broccoli	hsa-miR-217	MIMAT0000274	−2.53	0.06	*TRPS1*
	hsa-miR-362-3p	MIMAT0004683	1.84	0.06	Not predicted
	hsa-miR-590-5p	MIMAT0003258	−2.89	0.02	*ACTA2*, *APAF1*, *BMPR2*, *BTG2*, *C8orf44-SGK3/SGK3*, *CDC25A*, *CDK6*, *CFKN1A*, *CFL2*, *FAM3C*, *FAS*, *FASLG*, *GLCCI1*, *HIPK3*, *IL6R*, *JAG1*, *LRRFIP1*, *MARCKS*, *MTAP*, *NFIB*, *PDCD4*, *PELI1*, *PIK3R1*, *PRRG4*, *PTEN*, *RECK*, *RP2*, *SERPINB5*, *SESN1*, *SLC12A1*, *SLC16A10*, *SOCS5*, *SOD3*, *SOX5*, *SPRY1*, *SPRY2*, *TAGLN*, *TCF21*, *TGFBR2*, *TIMP3*, *TNF*, *TPM1*
	hsa-miR-612	MIMAT0003280	1.65	0.07	*AKT2*, *TP53*

a Each target mRNA was predicted using experimentally observed results from Ingenuity Pathway Analysis (IPA) (Qiagen, Hilden, Germany).

Gene Ontology predicted the biological pathways impacted by the intervention. In the carrot group, 20 significantly overrepresented pathways were found to be enriched by the predicted mRNA targets (**Supplementary Table 3**). In the broccoli group, 12 significantly overrepresented pathways were found to be enriched by the predicted mRNA targets (**Supplementary Table 4**). All pathways found to be enriched by the predicted mRNA targets for the broccoli group were also found to be enriched in the carrot group, with the addition of eight more pathways overrepresented in the carrot group.

### 3.4 Microbiota and miRNA

**Supplementary Figure 2** shows a heat map depicting the differentially expressed miRNAs and their correlations with the microbial taxa at the genus and species levels. MiR-186-5p, miR-590-5p, miR-769-5p, and miR-1253 were each correlated with changes in the abundance of three or more species.

## 4 Discussion

In adults, it has been shown that the gut microbiota quickly (within 1–2 days of intervention) changes and responds to diet composition (Leeming et al., 2019). However, for short-term interventions, these changes are often transient as the adult enterotypes are stable through interventions (David et al., 2014; Wu et al., 2011). In infants, it is known that bacterial colonization of the gut is in a development phase: the mode of delivery, type of feeding, and medication use impact early microbial composition (Robertson et al., 2019), and the gut microbiome continues to develop as infants are exposed to new signals such as environmental changes and the complementary diet (Deering et al., 2019). What has yet to be identified in the literature is how long it takes for dietary interventions to shape the microbial composition in infants, especially in light of the already maturing gut microbiome.

Prior literature has demonstrated that solid food introduction is associated with both higher diversity and observed number of species (i.e., bacterial load) (Koenig et al., 2011; Derrien et al., 2019). The present feasibility study found a trend towards a decrease in the Shannon index and a trend toward an increase in the observed number of species; more specifically, the number of more abundant organisms present in the microbiota of carrot-fed infants decreased, allowing for a concomitant increase in the less abundant organisms, which underscores a differential effect of the complementary foods on the gastrointestinal microbiota. While no significant differences were observed between groups for the unweighted UniFrac distance, that a significant group difference exists for the weighted UniFrac distance highlights that the relative abundance of some organisms did indeed differ between groups. With that, the carrot and broccoli groups have less similarity to one another than the carrot and broccoli groups have to the control, suggesting that dietary diversity has a great impact on the developing microbiota (Homann et al., 2021).

The relative abundance of *Streptococcus* increased in the carrot group compared to the control, which is consistent with select findings from an *in vitro* gastrointestinal digestion and fermentation study (Parkar et al., 2021). Our findings may further be explained by the carbohydrate composition of both carrots and broccoli. Carrots, as well as some fruits and berries, have pectin-rich cell wall polysaccharides (Parkar et al., 2021). Pectins are heteropolysaccharides that are structurally complex and composed of several linked polymers (Hamaker and Tuncil, 2014). Pectins are indigestible by human enzymes; however, the commensal bacteria within the gut are able to degrade them and produce short-chain fatty acids and other metabolites (Larsen et al., 2019). Pectins are known to stimulate the growth of microbial communities, thereby contributing to the diversity of the gut microbiota (Beukema et al., 2020). The differences in the structure and the availability of the carbohydrate sources across the carrot, broccoli, and control groups may have contributed to the group differences seen in bacterial taxa.

With respect to the miRNAs and their predicted mRNA targets, the mRNAs observed most often in regulating enriched pathways (determined by the PANTHER analysis) were *AKT2*, *MDM2*, *KRAS*, *PTEN*, *TP53*, *PIK3R1*, *MAPK12*, and *APAF1* in both broccoli- and carrot-fed infants. The differentially expressed miRNAs identified in the present study have been shown previously to be involved in cancer pathways in specific cancer models (Kim et al., 2019; Schetter et al., 2012); however, in a healthy infant, pathways with which the predicted mRNA may be involved include those related to signal transduction [e.g., p53 pathway, Fas signaling pathway, epidermal growth factor (EGF) receptor signaling, and fibroblast growth factor (FGF) signaling], and immune function (e.g., T-cell activation and the interleukin signaling pathway) (Levine et al., 2006; Smith-Garvin and Koretzky, 2009). Interestingly, in the current study, all predicted pathways found in the broccoli intervention were also found in the carrot intervention. Enriched pathways based on the predicted mRNA targets of the modulated miRNAs unique to the carrot intervention were all related to cell growth, homeostasis, and immune function [e.g., interferon-γ and interleukin signaling pathways, T-cell activation, transforming growth factor beta (TGF-β), platelet-derived growth factor (PDGF), and gastrin cholecystokinin (CCK) receptor pathways].

Although prior literature has not determined the effect of apiaceous and cruciferous vegetable consumption on miRNA expression in infants, rat cancer models investigating the chemoprotective effects of these vegetable families found similar results in relation to signaling, immune function, and inflammation, as well as the possible protective effects of these vegetable families against the development of colon cancer in both initiation (Arikawa and Gallaher, 2008) and post-initiation stages (Kim et al., 2019).

Taken together, complementary foods influence the composition of the infant gut microbiota and miRNA expression. Fecal miRNAs have the capacity to shape the gut microbiota and influence bacterial metabolism, specifically gene expression and gut microbial growth (Liu et al., 2016; Sarshar et al., 2020). Previous studies have examined the relationship between the microbiota and miRNA in the context of diseased states [i.e., colorectal cancer (Rubinstein et al., 2013), inflammatory bowel disease (Ji et al., 2018), and type 2 diabetes (Li et al., 2020)]. Diet has been shown to impact the expression of miRNA, which in turn is a contributor toward microbiota composition and host metabolism (Cui et al., 2017; Tarallo et al., 2022). In the present study, significant association and directionality were determined for the miRNAs differentially expressed by diet intervention and the genus and species levels of the taxa in the gut microbiota of healthy infants.

This was a feasibility study and, as such, had several limitations. The first was the sample size, which was sufficient for a proof-of-concept study, but not sufficiently powered to fully address between-subject variability. In this sample, the liquid diet was not homogenous: infants were allowed to be fed human milk, infant formula, or a combination for greater generalizability. In addition, while it was required that infants were not consuming fruits and vegetables over the study period, infant cereals were not restricted, and although the liquid diet or solid food consumption differed statistically among groups, the lack of homogeneity is a limitation. Future work that includes a larger sample size, a similar liquid diet, and no prior food introduction is needed to further explore the effects of singular food introduction on the infant gut microbiota and miRNA expression.

This short-term feeding of carrots or broccoli led to alterations in both the gut microbiota and miRNA expression, and while this speaks to the successful feasibility nature of this study, the results herein should be interpreted in the context of the small sample size. With respect to the changes observed in the microbiota, feeding carrots resulted in a trend towards a decreased Shannon index and tended to increase the number of observed ASVs compared to the control, with no differences between the broccoli and control groups for these indices. The relative abundance of *Streptococcus* increased in the carrot group compared to both the control and broccoli groups. Focusing on the changes observed in the miRNA expression, a higher number of miRNAs increased in the carrot group than in the broccoli group. Common to both vegetables, miR-217 and miR-590-5p decreased. MiR-590-5p predicted the most target mRNAs; the subsequent prediction of canonical pathways was for those involved in signal transduction. In addition, the differentially expressed miRNAs in the carrot group, but not in the broccoli group, also predicted the mRNA targets involved in pathways relating to immune function, cell growth, and homeostasis. That different/multiple pathways vital to health were predicted to be enriched in the carrot and broccoli groups may be a factor in why the total fruit and vegetable intake and consumption of a variety of vegetables has been shown to reduce disease risk (Aune et al., 2017; Stanaway et al., 2022). Variability in the quantity and types of fruits and vegetables consumed likely results in varied/complementary phytochemical intake, which in turn may be a mechanism for the beneficial effects of varied vegetable intake on health outcomes (Liu, 2003; van Breda and de Kok, 2018). The goal of this study was to assess the feasibility of short-term vegetable exposure in infants on changes in the gut microbiota and miRNA expression. That changes were observed as early as day 2 for microbiota outcomes and some miRNAs suggest that, in infants, bacterial communities and host gene expression change relatively rapidly in response to diet. However, limitations such as the small sample size and the lack of homogeneity among groups require that this study be repeated with a larger sample size to provide stronger evidence of the intervention effect and to confirm the length of intervention necessary. As such, this feasibility study with its RCT design may be used to inform the design of future studies evaluating the effects of diet interventions on the infant gut microbiota and miRNA expression.

## Data availability statement

The data presented in this study are deposited in the National Center for Biotechnology Information (NCBI): National Library of Medicine under BioProject PRJNA1047115, and Zenodo under DOI 10.5281/zenodo.10233296.

## Ethics statement

This study was approved by the University of Arkansas Institutional Review Board and was conducted in accordance with the local legislation and institutional requirements. Written informed consent for participation in this study was provided by the participants’ legal guardians.

## Author contributions

LEF: Data curation, Formal analysis, Visualization, Writing – original draft, Writing – review & editing. KB: Formal analysis, Methodology, Software, Visualization, Writing – review & editing. SPT: Formal analysis, Methodology, Writing – review & editing. KEB: Investigation, Writing – review & editing. SWP: Data curation, Formal analysis, Methodology, Resources, Software, Writing – review & editing. JKK: Conceptualization, Funding acquisition, Investigation, Methodology, Project administration, Resources, Writing – review & editing. JCT: Data curation, Formal analysis, Funding acquisition, Methodology, Resources, Supervision, Visualization, Writing – original draft, Writing – review & editing.
