# Supplementary material for: A short-term, randomized, controlled, feasibility study of the effects of different vegetables on the gut microbiota and microRNA expression in infants
Source: Front Microbiomes. 2024 Mar 1;3:1342464. doi: 10.3389/frmbi.2024.1342464 (PMC12993569; doi:10.3389/frmbi.2024.1342464)
Supplement: Supplementary file 1 [file Presentation_1.pptx]

## Slide 1
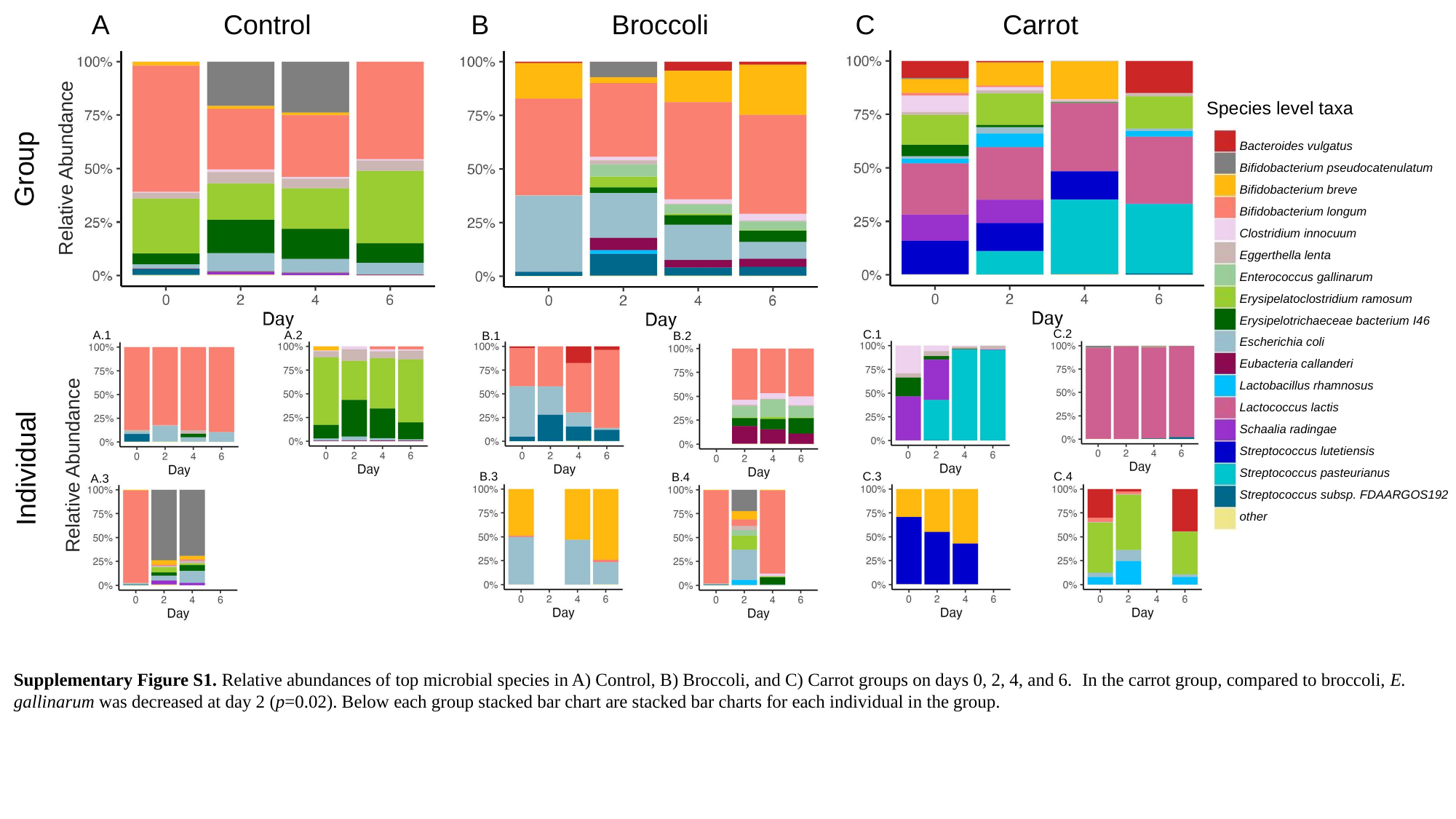

A	 Control B	 Broccoli 	 C	 Carrot
Species level taxa
Bacteroides vulgatus
Bifidobacterium pseudocatenulatum
Bifidobacterium breve
Bifidobacterium longum
Clostridium innocuum
Eggerthella lenta
Enterococcus gallinarum
Erysipelatoclostridium ramosum
Erysipelotrichaeceae bacterium I46
Escherichia coli
Eubacteria callanderi
Lactobacillus rhamnosus
Lactococcus lactis
Schaalia radingae
Streptococcus lutetiensis
Streptococcus pasteurianus
Streptococcus subsp. FDAARGOS192
other
Group
Relative Abundance
C.2
C.1
A.2
A.1
B.2
B.1
Individual
Relative Abundance
C.4
C.3
B.3
B.4
A.3
Supplementary Figure S1. Relative abundances of top microbial species in A) Control, B) Broccoli, and C) Carrot groups on days 0, 2, 4, and 6. In the carrot group, compared to broccoli, E. gallinarum was decreased at day 2 (p=0.02). Below each group stacked bar chart are stacked bar charts for each individual in the group.

## Slide 2
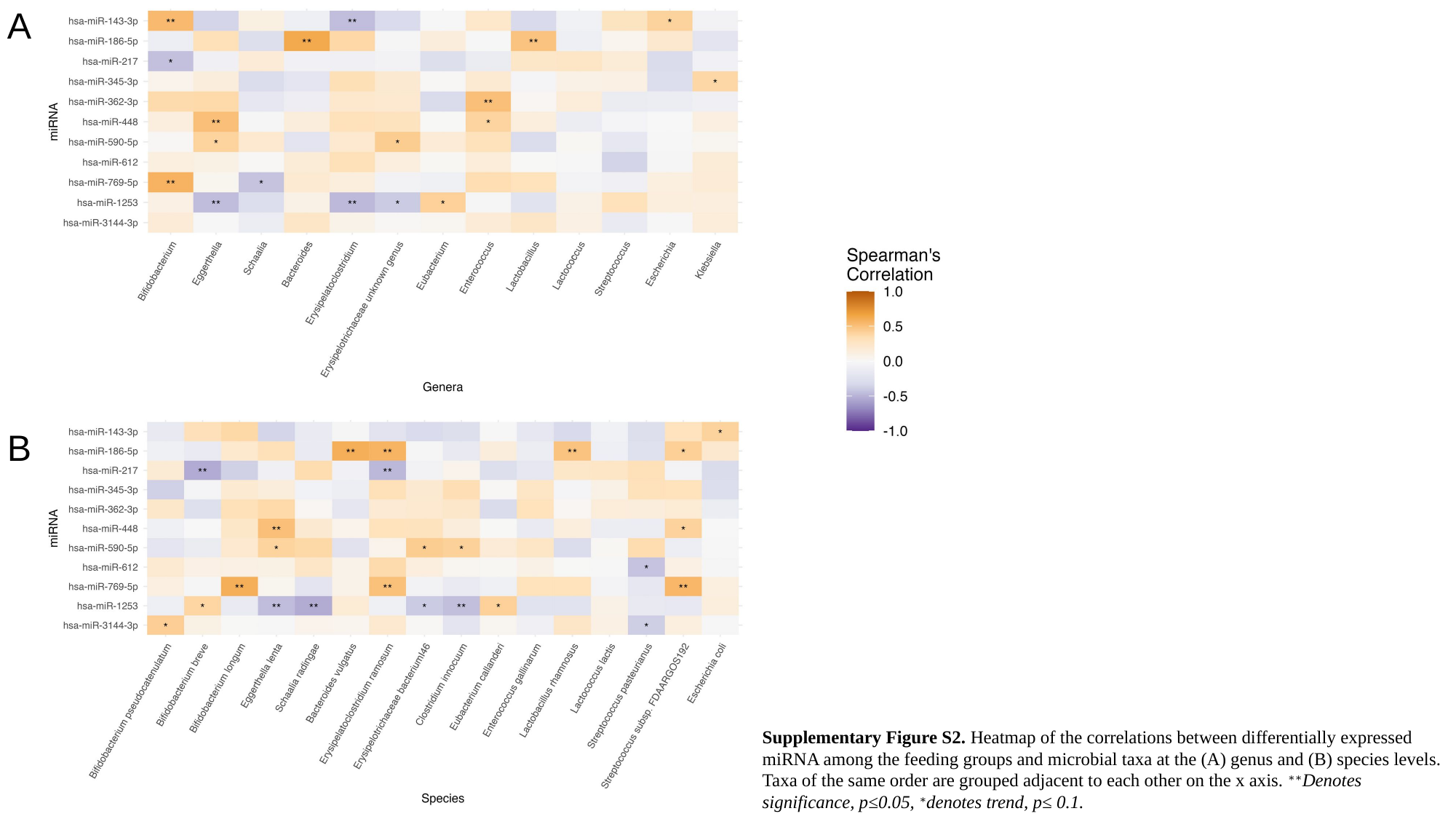

A
B
Supplementary Figure S2. Heatmap of the correlations between differentially expressed miRNA among the feeding groups and microbial taxa at the (A) genus and (B) species levels. Taxa of the same order are grouped adjacent to each other on the x axis. ∗∗Denotes significance, p≤0.05, ∗denotes trend, p≤ 0.1.
