# Supplementary material for: A short-term, randomized, controlled, feasibility study of the effects of different vegetables on the gut microbiota and microRNA expression in infants
Source: Front Microbiomes. 2024 Mar 1;3:1342464. doi: 10.3389/frmbi.2024.1342464 (PMC12993569; doi:10.3389/frmbi.2024.1342464)
Supplement: Supplementary file 2 [file Table_1.docx]

**Supplementary Table S1.** Participant Characteristics

|  | **All Groups**  **(N=11)** | **Control**  **(n=3)** | **Carrot**  **(n=4)** | **Broccoli**  **(n=4)** | ***p-*value** |
| --- | --- | --- | --- | --- | --- |
| Age at day 0, (mean ± SD^1^) | 6.14 ± 0.16 | 6.22 ± 0.07 | 6.12 ± 0.20 | 6.12 ± 0.18 | 0.64 |
| Male sex, N (%) | 5 (45) | 2 (67) | 1 (25) | 2 (50) | 0.77 |
| Liquid diet, N (%)  Human milk only  Infant formula only  Mixed fed (human milk and infant formula) | 7 (64)  3 (27)  1 (9) | 2 (67)  1 (33)  0 (0) | 2 (50)  1 (25)  1 (25) | 3 (75)  1 (25)  0 (0) | 0.73 |
| Exposure to solid food (cereal) prior to study, N (%) | 5 (45) | 2 (67) | 2 (67) | 1 (25) | 0.77 |

**Supplementary Table S2.** Individual participant characteristics and the corresponding relative abundance plots

| **Group** | **Identification in Figure 3 and Supplementary Figure S1** | **Liquid milk diet** | **Exposed to solid food (cereal) prior to study** |
| --- | --- | --- | --- |
| Control | A.1 | Human milk | No |
| Control | A.2 | Infant formula | Yes |
| Control | A.3 | Human milk | Yes |
| Broccoli | B.1 | Human milk | Yes |
| Broccoli | B.2 | Infant formula | No |
| Broccoli | B.3 | Human milk | No |
| Broccoli | B.4 | Human milk | No |
| Carrot | C.1 | Mixed fed | Yes |
| Carrot | C.2 | Infant formula | Yes |
| Carrot | C.3 | Human milk | No |
| Carrot | C.4 | Human milk | No |

**Supplementary Table S3.** Canonical pathways enriched by predicted mRNA targets in carrot fed infants

| **PANTHER Pathway^1^** | **N genes in reference genome dataset** | **N genes in dataset** | **N genes expected in dataset** | **Target mRNA identified in dataset^2^** | **Fold enrichment^3^** | ***p*-value^4^** |
| --- | --- | --- | --- | --- | --- | --- |
| P53 pathway feedback loops 1 | 7 | 2 | 0.02 | *MDM2, TP53* | 100 | 0.003 |
| p53 pathway feedback loops 2 | 51 | 7 | 0.14 | *AKT2, KRAS, MAPK12, MDM2, PIK3R1, PTEN, TP53* | 48.72 | <0.001 |
| Insulin/IGF pathway-protein kinase B signaling cascade | 40 | 5 | 0.11 | *AKT2, FOXO1, MDM2, PTEN, PIK3R1* | 44.37 | <0.001 |
| FAS signaling pathway | 34 | 4 | 0.1 | *AKT2, APAF1, FAS, FASLG* | 41.76 | <0.001 |
| Hypoxia response via HIF activation | 29 | 3 | 0.08 | *AKT2, PIK3R1, PTEN* | 36.72 | 0.002 |
| p53 pathway | 89 | 9 | 0.25 | *AKT2, APAF1, CDC25A, FAS, MDM2, PIK3R1, PTEN, SERPINB5, TP53* | 35.9 | <0.001 |
| Interferon-gamma signaling pathway | 30 | 3 | 0.08 | *MAPK7, MAPK12, SOCS5* | 35.5 | 0.002 |
| PI3 kinase pathway | 57 | 5 | 0.16 | *AKT2, FOXO1, KRAS, PIK3R1, PTEN* | 31.14 | <0.001 |
| p53 pathway by glucose deprivation | 23 | 2 | 0.06 | *AKT2, TP53* | 30.87 | 0.018 |
| Apoptosis signaling pathway | 125 | 7 | 0.35 | *AKT2, APAF1, BCL2, FAS, FASLG, TNF, TP53* | 19.88 | <0.001 |
| EGF receptor signaling pathway | 142 | 6 | 0.4 | *AKT2, KRAS, MAPK7, MAPK12, SPRY1, SPRY2* | 15 | <0.001 |
| FGF signaling pathway | 127 | 5 | 0.36 | *KRAS, MAPK12, SPRY1, SPRY2* | 13.98 | <0.001 |
| TGF-beta signaling pathway | 102 | 4 | 0.29 | *BMPR2, KRAS, MAPK12, TGFBR2* | 13.92 | 0.003 |
| Interleukin signaling pathway | 89 | 3 | 0.25 | *AKT2, IL6R, MAPK7* | 11.97 | 0.019 |
| T cell activation | 92 | 3 | 0.26 | *AKT2, KRAS, PIK3R1* | 11.58 | 0.019 |
| CCK receptor signaling map | 173 | 5 | 0.49 | *BCL2, FOXO1, MAPK7, PIK3R1, PTEN* | 10.26 | 0.002 |
| PDGF signaling pathway | 145 | 4 | 0.41 | *AKT2, KRAS* | 9.79 | 0.009 |
| Huntington disease | 152 | 4 | 0.43 | *ACTA2, AKT2, APAF1* | 9.34 | 0.009 |
| Angiogenesis | 181 | 4 | 0.51 | *AKT2, JAG1, KRAS, PIK3R1* | 7.84 | 0.016 |
| Inflammation mediated by chemokine and cytokine signaling pathway | 261 | 5 | 0.74 | *ACTA2, AKT2, KRAS, PTEN, SOCS5* | 6.8 | 0.009 |

^1^PANTHER (Protein ANalysis THrough Evolutionary Relationships) software used to determine predicted pathways based on the mRNA predictions (available at [www.pantherdb.org](http://www.pantherdb.org))

^2^Ingenuity Pathway Analysis (IPA) (Qiagen, Hilden, Germany)

^3^Fold enrichment was calculated via PANTHER software using the N genes in dataset divided by the N genes expected in dataset

^4^All p-values corrected for using the False Discovery Rate (FDR)

**Supplementary Table S4.** Canonical pathways enriched by predicted mRNA targets in broccoli fed infants

| **PANTHER Pathway^1^** | **N genes in reference genome dataset** | **N genes in dataset** | **N genes expected in dataset** | **Target mRNA identified in dataset^2^** | **Fold enrichment^3^** | ***p*-value^4^** |
| --- | --- | --- | --- | --- | --- | --- |
| FAS signaling pathway | 34 | 4 | 0.07 | *AKT2, APAF1, FAS, FASLG* | 55.05 | <0.001 |
| Hypoxia response via HIF activation | 29 | 3 | 0.06 | *AKT2, PIK3R1, PTEN* | 48.41 | 0.001 |
| p53 pathway | 89 | 8 | 0.19 | *AKT2, APAF1, CDC25A, FAS, PIK3R1, PTEN, SERPINB5, TP53* | 42.06 | <0.001 |
| p53 pathway by glucose deprivation | 23 | 2 | 0.05 | *AKT2, TP53* | 40.69 | 0.021 |
| p53 pathway feedback loops 2 | 51 | 4 | 0.11 | *AKT2, PIK3R1, PTEN, TP53* | 36.7 | <0.001 |
| Insulin/IGF pathway-protein kinase B signaling cascade | 40 | 3 | 0.09 | *AKT2, PIK3R1, PTEN* | 35.09 | 0.002 |
| PI3 kinase pathway | 57 | 3 | 0.12 | *AKT2, PIK3R1, PTEN* | 24.63 | 0.006 |
| Apoptosis signaling pathway | 125 | 6 | 0.27 | *AKT2, APAF1, FAS, FASLG, TNF, TP53* | 22.46 | <0.001 |
| Huntington disease | 152 | 4 | 0.32 | *ACTA2, AKT2, APAF1, TP53* | 12.31 | 0.006 |
| FGF signaling pathway | 127 | 3 | 0.27 | *AKT2, SPRY1, SPRY2* | 11.05 | 0.036 |
| EGF receptor signaling pathway | 142 | 3 | 0.3 | *AKT2, SPRY1, SPRY2* | 9.89 | 0.045 |
| Inflammation mediated by chemokine and cytokine signaling pathway | 261 | 4 | 0.56 | *ACTA2, AKT2, PTEN, SOCS5* | 7.17 | 0.035 |

^1^PANTHER (Protein ANalysis THrough Evolutionary Relationships) software used to determine predicted pathways based on the mRNA predictions (available at [www.pantherdb.org](http://www.pantherdb.org))

^2^Ingenuity Pathway Analysis (IPA) (Qiagen, Hilden, Germany)

^3^Fold enrichment was calculated via PANTHER software using the N genes in dataset divided by the N genes expected in dataset

^4^All p-values corrected for using the False Discovery Rate (FDR).
